# Comparison of the conventional and immersing powdered crude drugs (IPCD) methods for color and extraction of quantitative indicator ingredients in the Kampo formula decoction of daiokanzoto

**DOI:** 10.1186/s40780-023-00289-8

**Published:** 2023-07-01

**Authors:** Kazumasa Naruhashi, Natsumi Amaike, Karen Shiraishi, Sumire Sato, Chiho Uryuzu, Yui Saito, Narumi Tsue

**Affiliations:** Education and Research Center for Pharmacy Practice, Faculty of Pharmaceutical Sciences, Doshisha Women’s College of Liberal Arts, Kodo Kyotonabe-shi, Kyoto, 610-0395 Japan

**Keywords:** Kampo, Kampo formula decoction, Conventional method, IPCD, Daiokanzoto, Rhubarb, glycyrrhiza, Sennoside a, Glycyrrhizic acid

## Abstract

**Aim:**

The immersing powdered crude drugs (IPCD) method is a quick and simple method for preparing decoctions. Here, the conventional and IPCD methods were compared for the color and extraction of quantitative indicator ingredients in the daiokanzoto decoction solution, and the suitability of the IPCD method was assessed.

**Methods:**

The color of decoction solutions was visually observed, and the Commission Internationale de L’éclairage (CIE) L*a*b*color parameters were measured using conventional and IPCD methods. The extracted amounts of sennoside A and glycyrrhizic acid, which are quantitative indicator ingredients of rhubarb and glycyrrhiza, respectively, were quantified.

**Results:**

Using both methods, the decoction solution colors were strong for rhubarb alone and daiokanzoto but weak for glycyrrhiza alone. The color change of daiokanzoto was thought to be primarily caused by rhubarb alone. The L*a*b* values of the decoction solution determined by the IPCD method were comparable to those determined by the conventional method (60 min). Using the conventional method, sennoside A and glycyrrhizic acid were mostly extracted in 10 and 30 min, respectively. Using the IPCD method, both sennoside A and glycyrrhizic acid were fully extracted in 2 min. The IPCD method yielded significantly more sennoside A and glycyrrhizic acid (2 times and 1.5 times, respectively) than the conventional method (60 min).

**Conclusion:**

The IPCD method was found to be comparable to the conventional method in terms of the color, and using IPCD method, the same or greater amounts of quantitative indicator ingredients of crude drugs in the decoction of daiokanzoto compared to the conventional method. It was suggested that there are limitations to assessing the equivalence of decoctions from decoction color. The IPCD method may be a useful method although it is prudent to use the IPCD method for Kampo formula decoction in clinical practice with a certain degree of caution.

**Supplementary Information:**

The online version contains supplementary material available at 10.1186/s40780-023-00289-8.

## Background

Kampo had been independently developed in Japan Edo era from ancient Chinese medicine transported from mainland China in 7th century. Kampo formulas have been then adapted and modified to fit the Japanese climate, culture, and traditions, and it is now widely used as a traditional medicine in Japan [1].

The Japanese Pharmacopoeia contains a listing of crude drugs and Kampo formulas. In 1960, the National Health Insurance drug price list included the crude drugs listed in the Japanese Pharmacopoeia. Kampo formulas were first listed in the Japanese Pharmacopoeia in 1967, and currently, 294 Kampo formulations for prescription are currently approved, and 148 of which are listed as their extracts on the Japanese Pharmacopoeia 18th Edition (JP18) and its supplement 1 as of December 2022 [2]. Not only the approved Kampo formulations but also other Kampo formulas are widely used as both prescription and over-the-counter drugs. In addition to its traditional use, it is also being applied to the treatment of dementia and the reduction of chemotherapy-related side effects.

The majority of Kampo medicines used in Japan today are “extract granules” [3]. Extract granules are convenient to carry and have a mild taste (especially bitterness) and aroma, making them suitable for oral consumption. Flavor and aroma are thought to be important for the efficacy of decoctions in particular. The conventional decoction method is usually preferred for this purpose. However, the conventional decoction method is time-consuming. Patients need 30 to 60 min to prepare the Kampo formula decoction for boiling [1]. Furthermore, the conventional decoction method is very inconvenient and adds to the burden of patients. Therefore, to save time and effort, Fueki et al. developed the immersing powdered crude drugs (IPCD) method as a quick, simple, and high-yielding method for preparing decoctions [4–7]. The IPCD method is a newly developed decoction method in which powdered crude drugs are immersed in boiling water to extract components. The IPCD method is based on the “Zhu san Fa (shasan-hou),” which was popular in China mainly in the Song Dynasty, and boils powdered crude drugs for one dose [8, 9]. The IPCD method uses a wine carafe to separate the immersion liquid from the muddy crude drug residue, making it a simpler and more practical preparation method. While the conventional decoction method takes half an hour or more, the IPCD method takes less than 5 min, greatly reducing the preparation time. Although the validity of the IPCD method has been reported in several studies, the available data is still limited.

Daiokanzoto is a Japanese Kampo formula composed of rhubarb (the rhizome of *Rheum palmatum Linneá*, *Rheum tanguticum* Maximowicz, *Rheum officinale* Baillon, *Rheum coreanum* Nakai or their interspecific hybrids [*Polygonaceae*]), and glycyrrhiza (the root and stolon, with [unpeeled] or without [peeled] the periderm, of *Glycyrrhiza uralensis* Fisher or *Glycyrrhiza glabra* Linneá [Leguminosae]). It is used in the treatment of constipation and its related symptoms, such as heavy head, hot flashes, eczema/dermatitis, acne, anorexia, abdominal bloating, abnormal intestinal fermentation, and hemorrhoids [10, 11].

Color is an important quality parameter in wines, being one of the first evaluated attributes during wine tasting. Wine color is the result of a complex mixture of pigments, which changes over time, due to chemical reactions between the pigments and also with oxygen and other wine components [12]. Therefore, it is thought that color can be one of the parameters to evaluate the Kampo formula decoction solution.

In this study, the conventional and IPCD methods were compared for the color and extraction of quantitative indicator ingredients in the daiokanzoto decoction solution, and the suitability of the IPCD method was assessed.

## Methods

### Materials

Crude drugs; rhubarb (the rhizome of *Rheum palmatum Linneá*, *Rheum tanguticum* Maximowicz, *Rheum officinale* Baillon, *Rheum coreanum* Nakai or their interspecific hybrids [*Polygonaceae*]) and glycyrrhiza (the root and stolon, with [unpeeled] or without [peeled] the periderm, of *Glycyrrhiza uralensis* Fisher or *Glycyrrhiza glabra* Linneá [Leguminosae]), which are registered and specified as crude drugs in the JP18, were purchased commercially from Tochimoto Tenkaido Co. (Osaka, Japan). TSUMURA Daiokanzoto Extract Granules for Ethical Use were purchased from Tsumura & Co. The daily dose of daiokanzoto consisted of a combination of rhubarb (4.0 g) and glycyrrhiza (2.0 g) according to regimen #2 in the JP18 [2].

### Decoction preparation using the conventional method and sampling

The conventional method of decoction preparation was modified the method previously documented [4, 7, 13] and used an electric Kampo formula decoction heater pot (My New Mycon Torobi-ranran, EKSA-10, Tochimoto-Tenkaido Co.). Commercially available crude drugs were used as it is, without any treatment. The mixture of crude drugs or one of each crude drug (the daily dose: rhubarb 4.00 g, glycyrrhiza 2.00 g) was added to the pot of boiling distilled water (500 mL) (time 0 min). The decoction continued to boil on the electric heater for 30 min, after which the heater was turned off and the decoction was set aside for 30 min. At 2, 5, 10, 20, 30, and 60 min, the decoction solution was collected without adding water. The amount of water that evaporated was calculated by comparing the weights before and after the experiment.

### Decoction preparation using the IPCD method and sampling

The IPCD method of decoction preparation has been previously described [4–6]. Each crude drug (approximately 40 g) was milled once for 1 min in an electric tabletop mill (Y-308B, Yamamoto Denki, Fukushima, Japan). Without sieving, the powder of milled crude drug was used for the IPCD method.

The powder mixture of crude drugs or that of each of the crude drugs (1/3 of the daily dose: rhubarb 1.33 g, glycyrrhiza 0.667 g) was placed in a wine carafe (0.25 L; Luminarc, Dubai, UAE), with the size and structure as reported by Fuek et al. After adding 150 mL of boiled distilled water, the suspension was vigorously stirred for 20 Sect. (2 strokes/sec). Following the 20-sec stirring (time 0 min), it was incubated for 4 min to allow the murky residue of the crude drug powder to precipitate. The immersion liquid was gently decanted into a beaker using a tea strainer. At 2 and 4 min, the decoction solution was collected.

### Extraction from daiokanzoto extract granules

Daiokanzoto extract granules (one pack = 1/3 of the daily dose) were dissolved in 150 mL of 50% methanol in a beaker and homogenized (Smurt: NR-50 M, Microtec Co., Ltd., Chiba, Japan).

### Spectrophotometric analysis of decoction solution

The decoction solution was centrifuged at 1,900 *g* for 30 min to obtain the supernatant for measuring color and transparency.

The spectrophotometer (Nippon Denshoku, Tokyo, Japan) was used to measure the Commission Internationale de L’éclairage (CIE) L*a*b*color parameters of the supernatant sample. L*a*b* is a color space defined by the Commission Internationale de L’éclairage (CIE). The lightness value, L*, assigns 0 to black and 100 to white. The a* axis is relative to the green–red opponent colors, with negative values pointing toward green and positive values pointing toward red. The b* axis represents the blue–yellow opponents, with negative numbers pointing toward blue and positive numbers pointing toward yellow [14].

### Quantification of quantitative indicator ingredients

According to JP18, sennoside A and glycyrrhizic acid are quantitative indicator ingredients of rhubarb and glycyrrhiza, respectively. The quantitative indicator ingredients were quantified using high-performance liquid chromatography (HPLC). As standard marker compound reagents, sennoside A (for the Japanese Pharmacopoeia Crude Drugs Test [for TLC]) and glycyrrhizic acid Standard (for HPLC) were purchased from Fujifilm Wako Chemicals Corporation (Tokyo, Japan). The authors should add the company name, city, and country names of these reagents.

The decoction solution samples were centrifuged at 15,780 *g* for 10 min to obtain the supernatant. The supernatant was subsequently filtrated through a membrane filter (Cosmonice Filter [W] [0.45 μm], Nacalai Tesque, Inc., Kyoto, Japan). The filtrate was then subjected to the HPCL system.

The LC-20 A Modular HPLC System (Shimadzu Corporation, Kyoto, Japan) was used to perform HPLC. It consisted of an LC-20AD liquid pump, an SIL-20 A autoinjector, an SPD-20 A UV spectrophotometric detector, and an OTC-20 A column oven. The HPLC conditions adhered to the JP18 [2] with few modifications, as summarized in Table 1.

**Table 1:**
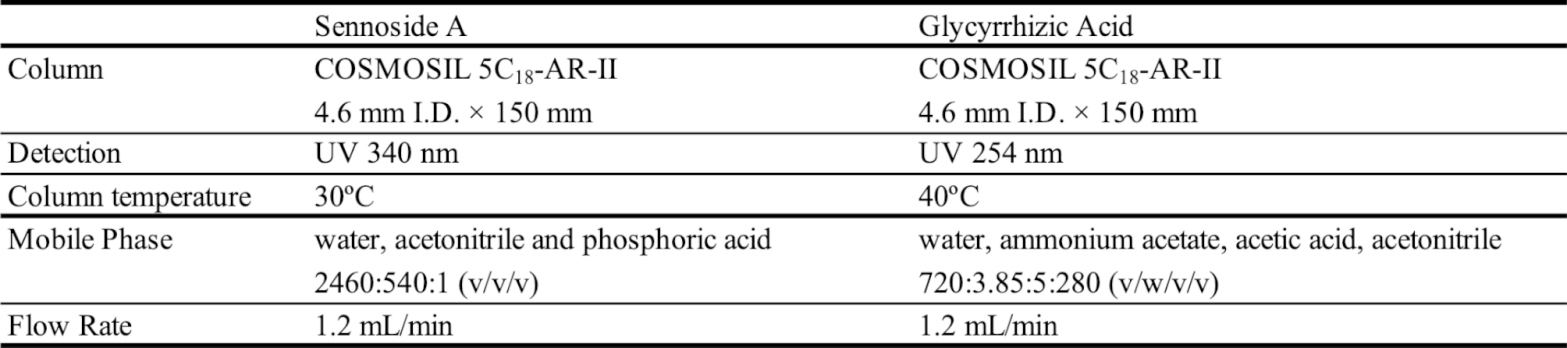
HPLC conditions COSMOSIL 5C_18_-AR-II were purchased from Nacalai Tesque, Inc., Kyoto, Japan

### Statistical analysis

All data are expressed as means ± SEM, and statistical analysis was performed by using Student’s *t*-test. A difference between means was considered to be significant when the *p*-value was less than 0.05.

## Results

### Spectrophotometric analysis of decoction solution

#### Rhubarb-alone (Fig. 1A)

**Fig. 1:**
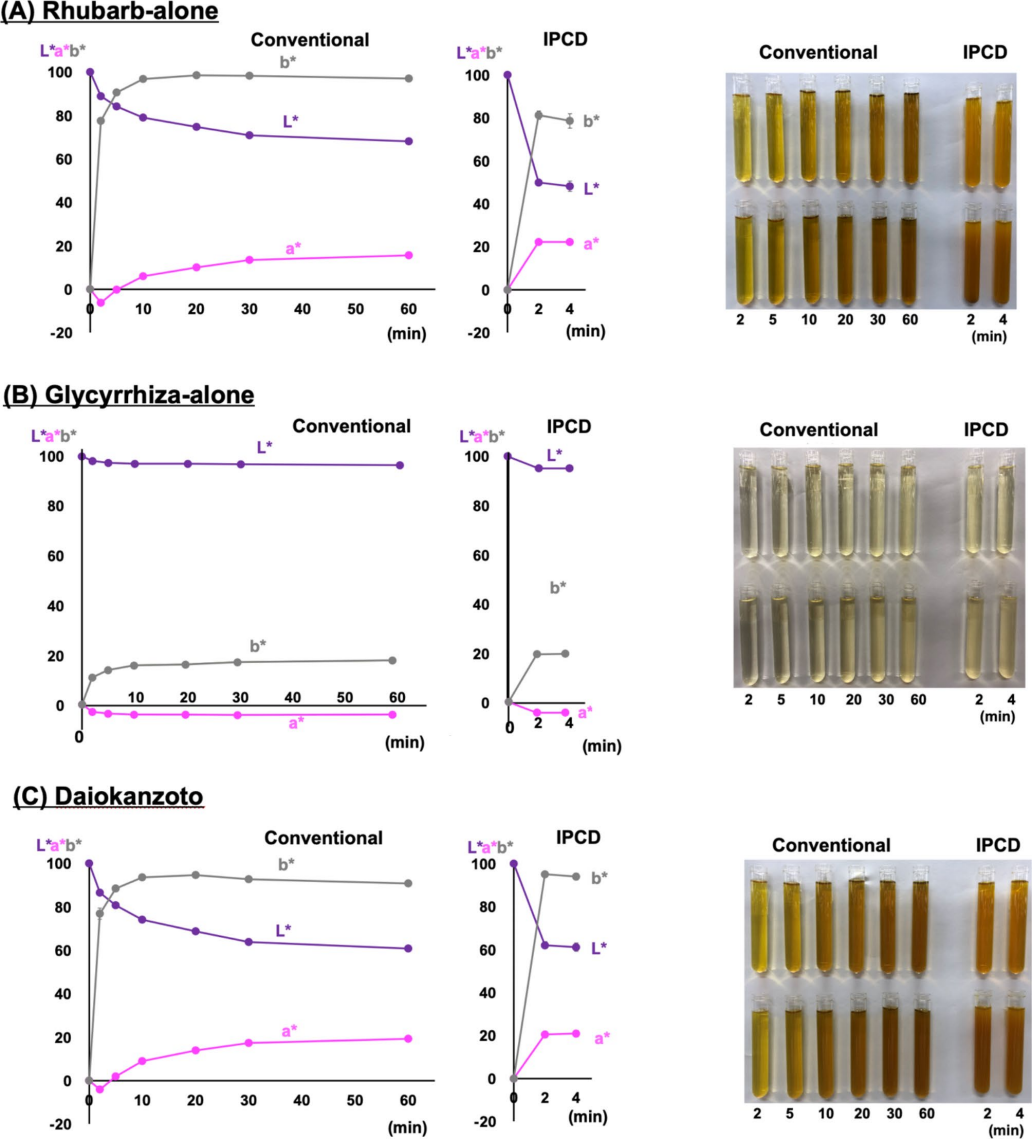
The color changes of daiokanzoto decoction solutions using the conventional and IPCD methods **A**: rhubarb-alone, **B**: glycyrrhiza-alone, **C**: daiokanzoto Left graphs: Commission Internationale de L’éclairage (CIE) L*a*b* color parameters of the supernatant samples (mean ± SEM, n = 4) Right photos: Upper: supernatant (after centrifugation); Lower: suspension (before centrifugation)

Using the conventional decoction method, it was visually confirmed that the decoction solution began to change color immediately after the start, darkened rapidly after 10 min, and darkened slightly thereafter. Consistent with visual observation, the a* and b* values of the color tone increased from 10 to 20 min. The value of L* decreased relatively quickly between 10 and 20 min and decreased gradually thereafter.

By the IPCD method, the decoction solution began to change color immediately after the start. Consistent with visual observation, the a* and b* values of the color tone reached their maximum values within 2 min.

The color of the decoction solution obtained using the IPCD method was slightly darker than that obtained using the conventional method. Furthermore, the values of a* was higher and b* was lower, while the value of L* was lower.

#### Glycyrrhiza-alone (Fig. 1B)

Using the conventional decoction method, it was visually confirmed that the decoction solution began to change color immediately after the start, but the color was extremely light and remained so even after 60 min. As for the color tone, b* displayed a certain value, but the value of b* remained unchanged, which visually corresponds to a slight yellowish tint. The value of a* was extremely low, consistent with the fact that visual confirmation of redness was nearly impossible. The value of L* decreased slightly after 5 min but remained constant thereafter.

Using the IPCD method, the decoction solution also began to take on color shortly after the start, but it was extremely light and remained so even after 4 min. As for the color tone, b* displayed a certain value, but there was no difference between 2 and 4 min, which visually corresponds to a slight yellowish tint. The value of a* was extremely low, consistent with the fact that visual confirmation of redness was nearly impossible.

The IPCD method produced a slightly darker decoction solution than the conventional method, but overall, the color was extremely light, resulting in small absolute values of a* and b* as well as a value of L* close to 100.

#### Daiokanzoto (Fig. 1C)

The changes in the decoction solution were very similar to those observed with rhubarb alone using conventional and IPCD methods, but the color of the decoction solution obtained using the conventional and IPCD method were very similar. The values of a*, b* and L* were comparable.

### Extraction amount of sennoside A

**Fig. 2:**
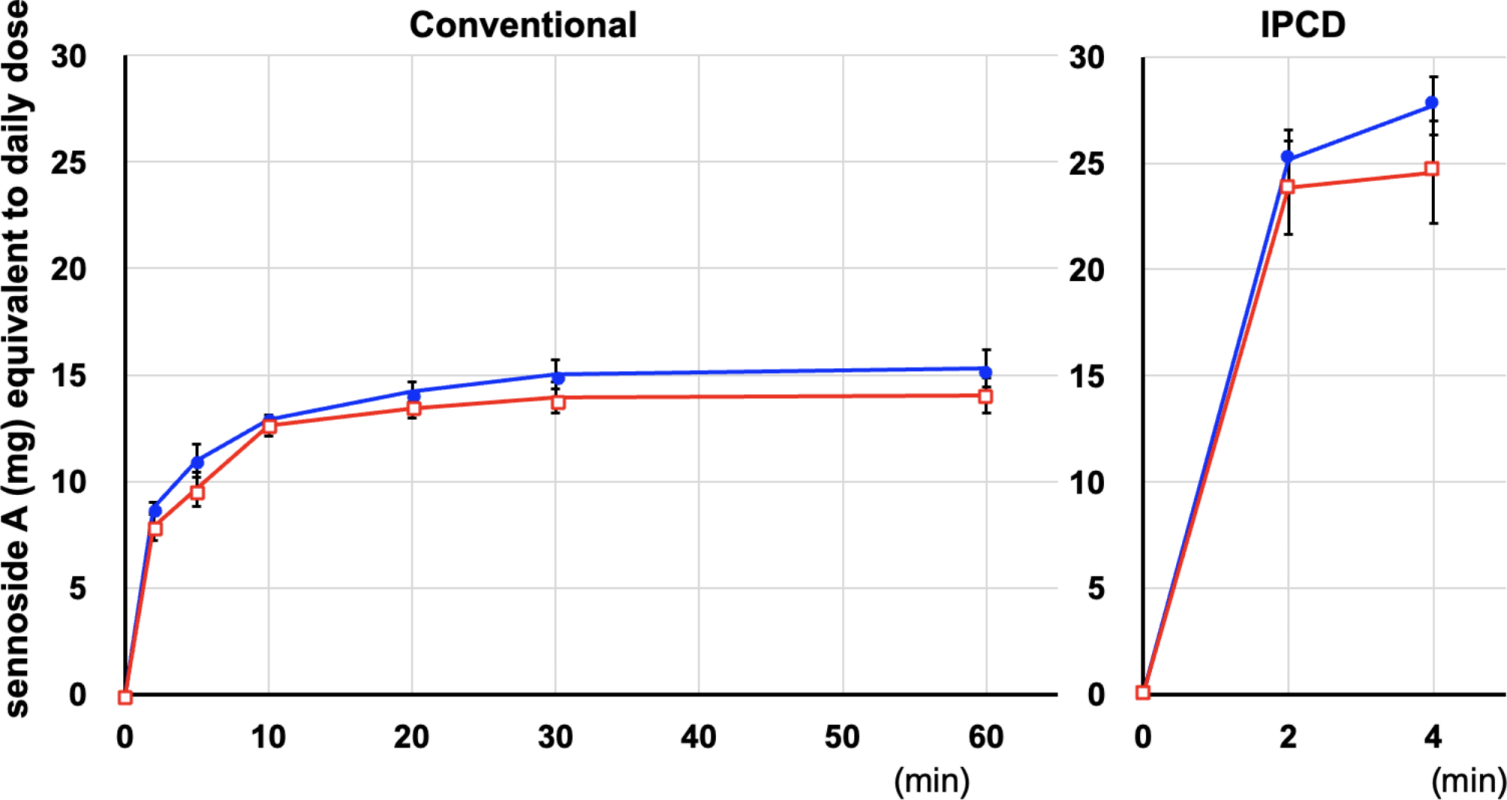
The extraction time course of sennoside A in the decoction solution from rhubarb alone and daiokanzoto using the conventional and IPCD methods Each point corresponds to the mean ± SEM of four independent experiments □rhubarb alone ●daiokanzoto

The extraction time course of sennoside A in the decoction using the conventional method was comparable between rhubarb alone and daiokanzoto. Sennoside A was extracted quickly from 2 to 10 min, then gradually increased and became constant after 20 min. The amount of sennoside A extracted at 60 min was comparable between rhubarb alone (15.3 ± 0.9 mg) and daiokanzoto (14.1 ± 0.8 mg) (Fig. 2).

Using the IPCD method, the extraction of sennoside A from rhubarb alone and daiokanzoto was completed in 2 min and increased slightly over the next 4 min. At 4 min, the amount of sennoside A from rhubarb alone (27.7 ± 1.4 mg) was slightly higher than that from daiokanzoto (24.6 ± 2.4 mg), even though no statistical significance was observed (Fig. 2).

**Fig. 3:**
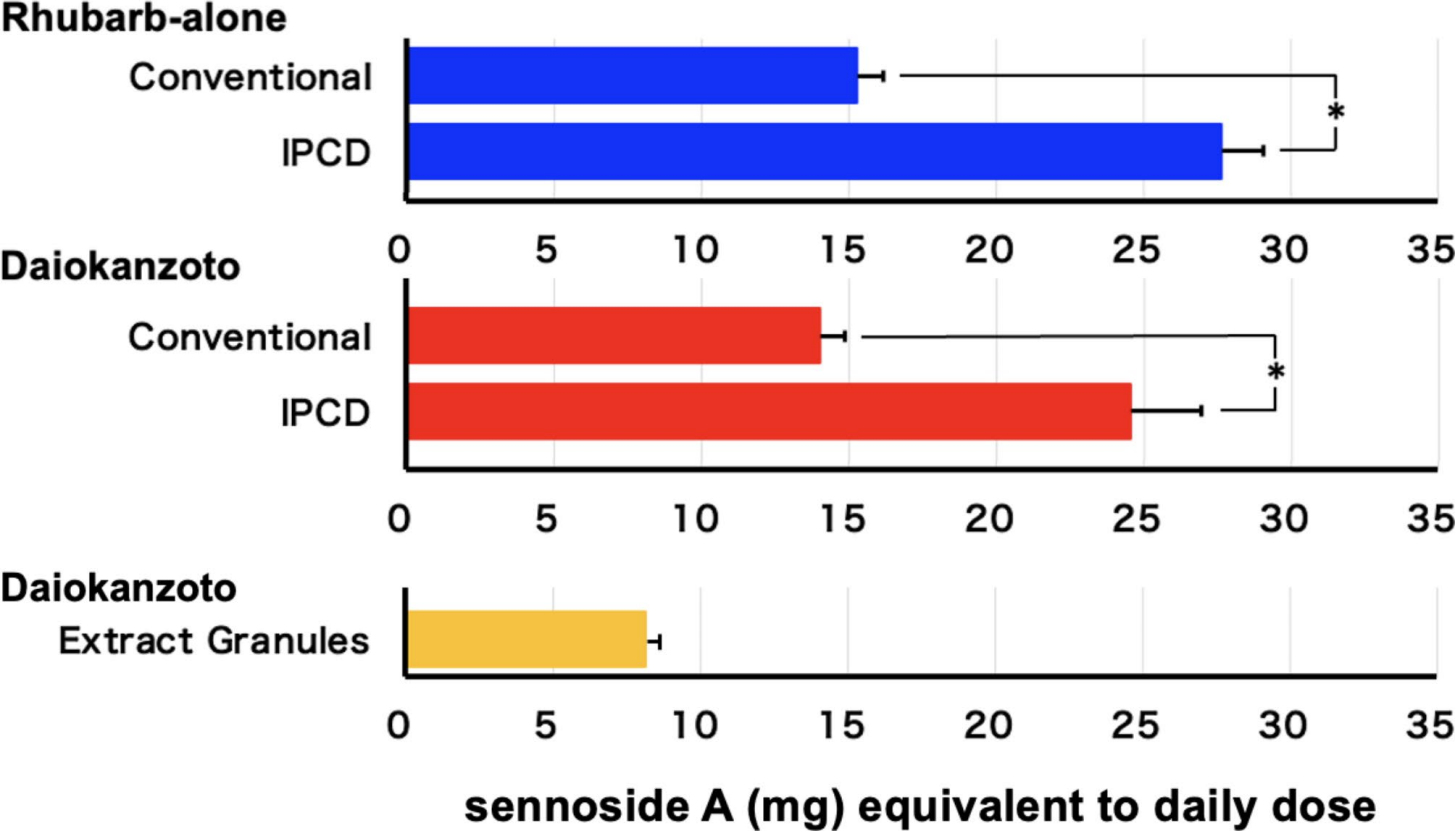
Comparison of the amounts of sennoside A extracted from rhubarb alone and daiokanzoto at the end of the conventional (60 min) and IPCD (4 min) methods Each bar corresponds to the mean ± SEM of four independent experiments * *p* < 0.05 (t-test)

The amount of sennoside A extracted at the end of the IPCD method (4 min) was significantly higher than that extracted at the end of the conventional decoction method (60 min) in both rhubarb alone and daiokanzoto (Fig. 3).

The sennoside A content in the extract granules of the commercial extract was considerably lower than in both decoctions but still within the limits of the JP18 (not less than 7 mg) (Fig. 3).

### Extraction amount of glycyrrhizic acid

**Fig. 4:**
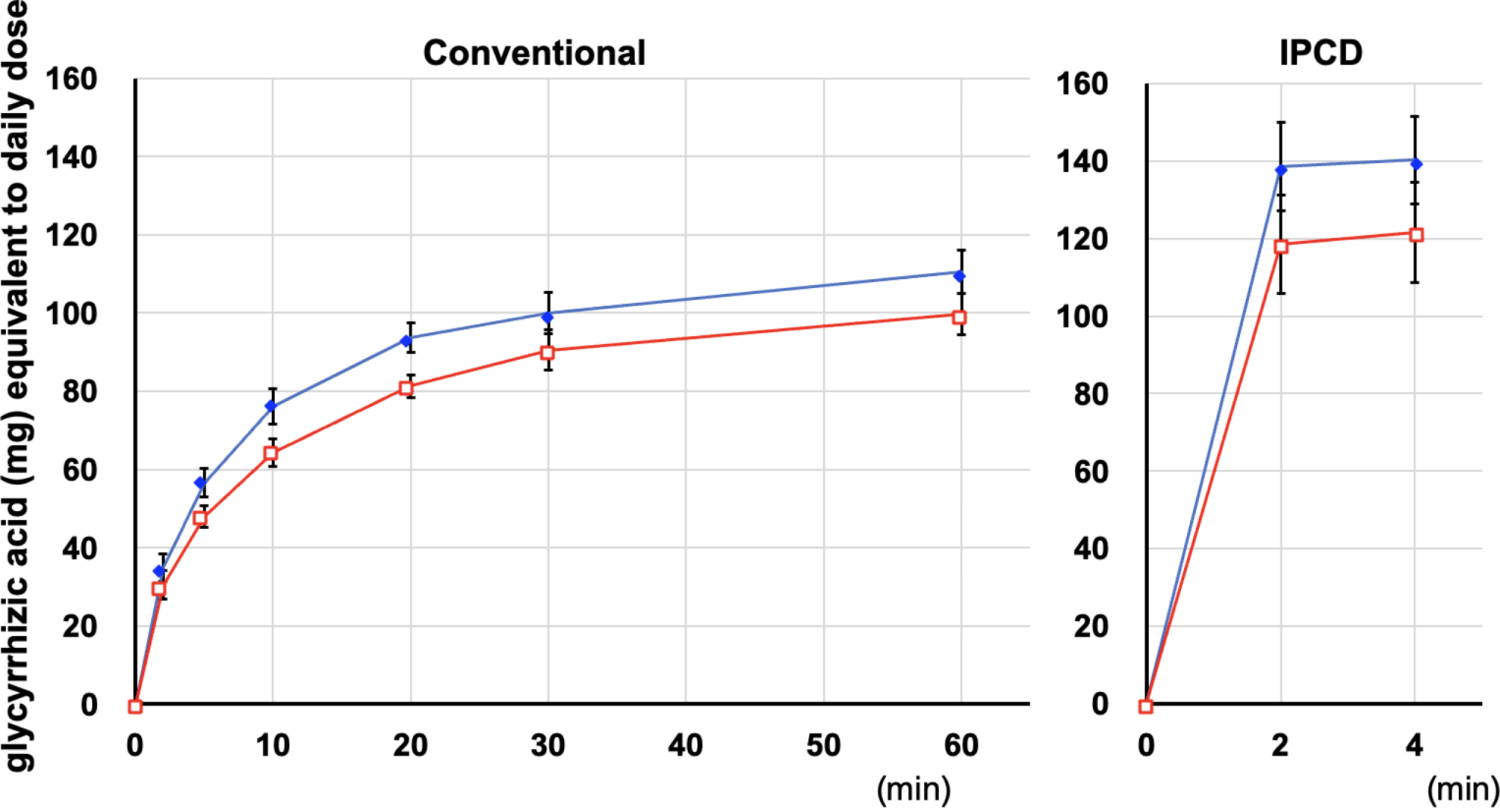
The extraction time course of glycyrrhizic acid in the decoction solution from glycyrrhiza alone and daiokanzoto using the conventional and IPCD methods Each point corresponds to the mean ± SEM of four independent experiments ◆glycyrrhiza alone □daiokanzoto

The extraction time course of glycyrrhizic acid in decoction using the conventional decoction method was comparable between glycyrrhiza alone and daiokanzoto. Glycyrrhizic acid was extracted gradually over 30 min and then gradually increased. The amount of glycyrrhizic acid extracted from glycyrrhiza alone was slightly higher than that extracted from daiokanzoto, but there was no statistical difference at 60 min between glycyrrhiza alone (110 ± 6 mg) and daiokanzoto (100 ± 6 mg) (Fig. 4).

Using the IPCD method, the extraction of glycyrrhizic acid from glycyrrhiza alone and daiokanzoto was completed in 2 min and increased slightly over the next 4 min. At 4 min, the amount of glycyrrhizic acid from glycyrrhiza alone (140 ± 11 mg) was slightly higher than that from daiokanzoto (132 ± 7 mg), even though no statistical significance was observed (Fig. 4).

**Fig. 5:**
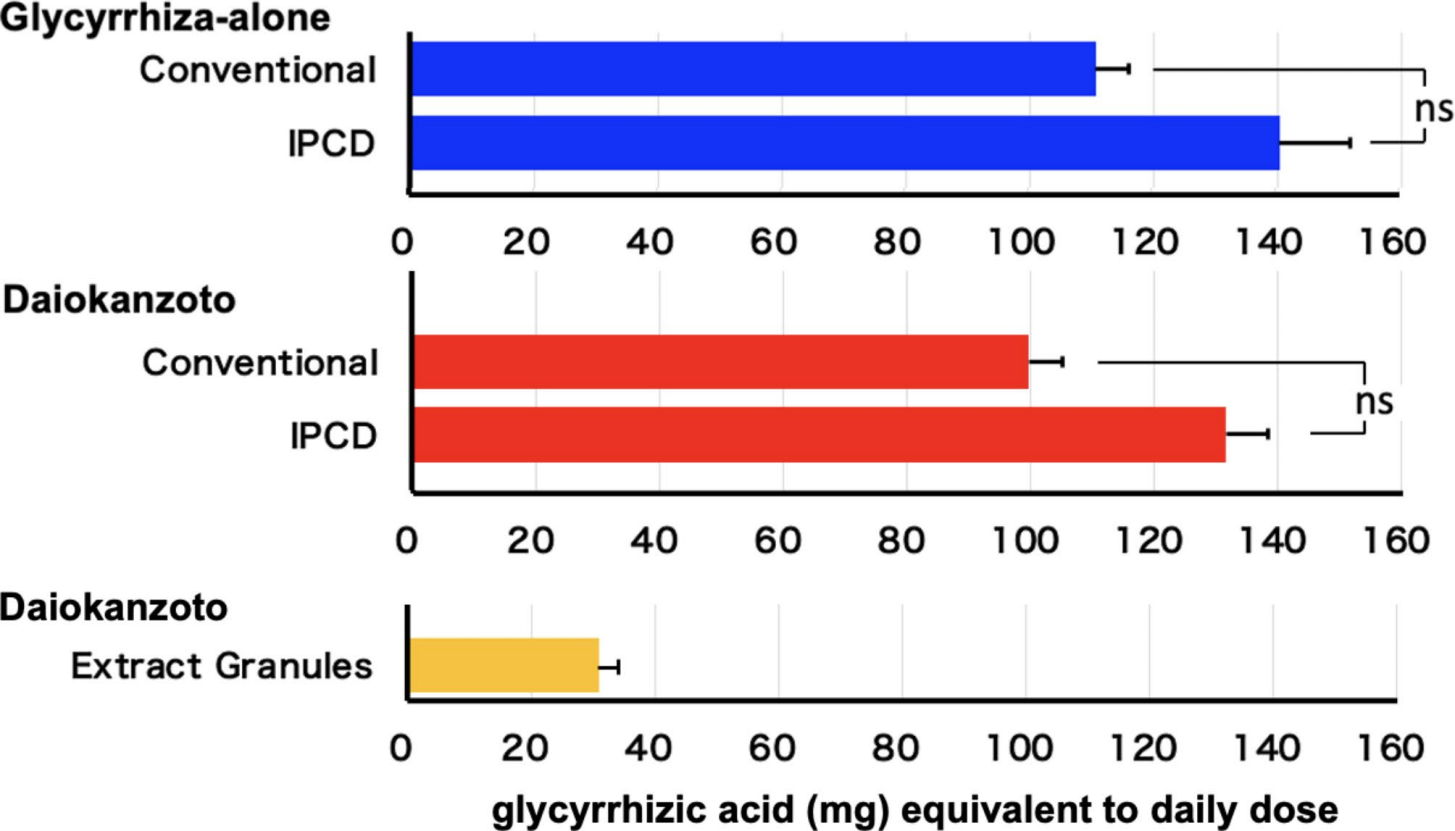
Comparison of the amounts of glycyrrhizic acid extracted from glycyrrhiza alone and daiokanzoto at the end of the conventional (60 min) and IPCD (4 min) methods Each bar corresponds to the mean ± SEM of four experiments ns: not significant (*p* > 0.05) (*t*-test)

The amount of glycyrrhizic acid extracted at the end of the IPCD method (4 min) was higher than that extracted at the end of the conventional decoction method (60 min) in both glycyrrhiza alone and daiokanzoto, but the differences were not significant (Fig. 5).

The glycyrrhizic acid content in the extract granules of the commercial extract was considerably lower than in both decoctions but still within the limits of the JP18 (14 ~ 42 mg) (Fig. 5).

## Discussion

Kampo formula decoction requires time and effort to boil, which places a significant burden on patients. To reduce preparation time, the immersion crude drug powder (IPCD) method was developed. While the conventional method takes 30 min, the IPCD method takes only 4 min, greatly reducing the preparation time. Although some studies claim that the IPCD extraction method is as effective as the conventional method, there is still room for clarification [4–7]. In addition, because the Kampo formula decoction is a mixture of known and unknown components/substances, it is impossible to compare the extracted amounts of all components/substances by the conventional and IPCD methods. Therefore, we used the color of the decoction solution as an objective index [14].

We chose daiokanzoto because it is one of the most popular Kampo formulas in Japan, both as a prescription medicine and as an over-the-counter drug [3].

Daiokanzoto is composed of two crude drugs, rhubarb, and glycyrrhiza, and is used to treat constipation and symptoms associated with constipation. Sennoside A and glycyrrhizic acid are the quantitative indicator ingredients specified in the JP18 [2], as well as the active ingredients of daiokanzoto. Sennoside A has a laxative effect [15], while glycyrrhizic acid has an anti-inflammatory effect [16] and is thought to relieve painful defecation and abdominal pain associated with constipation [10, 11].

The color of the decoction solution began to change after adding chopped crude drugs (daiokanzoto, rhubarb alone, and glycyrrhiza alone) to boiling purified water in the conventional decoction method, and it became darker immediately (5 min), then slightly darker. There was almost no color change between 30 and 60 min. The values of a* and b* increased while L* decreased over time, which coincided with the increase in redness and yellowness, and visual darkness. This color change (visual observation, values of L*a*b*) was strong for rhubarb alone and daiokanzoto but weak for glycyrrhiza alone. The color change of daiokanzoto was thought to be primarily caused by rhubarb alone.

In the IPCD method, a visual observation confirmed that the color of the decoction solution began to change after adding powdered crude drugs (daiokanzoto, rhubarb alone, and glycyrrhiza alone) to boiled purified water, with almost no change between 2 and 4 min. Even after 2 min, the values of a* and b* were sufficiently high, while L* was low, consistent with the strong visual redness, yellowness, and darkness. These values were comparable to those of the decoction solution obtained using the conventional method (60 min).

Using the conventional method, sennoside A (from daiokanzoto and rhubarb alone) was mostly extracted in 10 min, with a little increase thereafter, whereas glycyrrhizic acid (from daiokanzoto and glycyrrhiza alone) was extracted in 30 min, and slightly increased in 60 min. Using the IPCD method, both sennoside A (from daiokanzoto and rhubarb alone) and glycyrrhizic acid (from daiokanzoto and glycyrrhiza alone) were fully extracted in 2 min, with a little increase even in 4 min. The IPCD method yielded considerably more sennoside A (2 times), and more glycyrrhizic acid (1.5 times) than the conventional method (60 min). Previously, the IPCD method was reported to have a higher extraction efficiency for sennoside A (approximately 1.1–1.2 times) [7]. Strictly speaking, in the conventional decoction method described [4, 7, 13], Kampo formula decoction is started by adding crude drugs to cold water and then boiled for 30 min. However, in this study, the water was boiled and then the crude drugs were added and heated for 30 min since the time it takes for the cold water to become boiled water varies greatly depending on the temperature of the cold water and the room temperature of the laboratory. The time boiled at higher temperature was longer in this study than the previous study [7]. It was reported that sennoside A in decoction solution from rhubarb was decomposed and the amount was decreased to 81% by heating for 40 min [17]. Therefore, the more prominent conventional-to-IPCD ratio of sennoside A in this study may be due to the increased sennoside A decomposition by the longer heating time in this study. In contrast, it has been reported that the decoction rate of glycyrrhizic acid from another Kampo formula, ryokeikansoto, and other quantitative indicator ingredients, including those with high heat stability, is high [6]. The IPCD method is supported as a method for extracting many components from crude drugs more efficiently. Due to the high extraction efficiency of the IPCD method, the extraction of an excessive amount of sennoside A from daiokanzoto may cause diarrhea as an excessive laxative effect. In addition, the extraction of an excessive amount of glycyrrhizic acid may result in the side effects of pseudoaldosteronism, such as hypertension accompanied by hypokalemia, a heavy head feeling associated with increased blood pressure, and weakness of the extremities due to hypokalemic myopathy. However, in the Kampo formula of daiokanzoto prescribed by JP18, these concerns are unnecessary. It is preferable to prescribe a reduced dose of the overlapping crude drugs to patients who are taking additional Kampo simultaneously. Moreover, this has the added advantage of reducing the wasteful utilization of crude drug resources.

Regarding the color tone of the decoction solution by the both decoction methods, it was evaluated as being comparable overall by visual observation and L*a*b*color parameters, but there were slight differences. Sennoside A is a pigment compound that has a bianthrone glycoside structure and exhibits a yellow color. Therefore, it could be conceivable that the amount of sennoside might be evaluated by color tone evaluation. In this study, Rhubarb alone by the IPCD method showed a lower b* value, meaning duller color, which may be related to the higher amount of sennoside A extracted. But there was no difference in the b* values in the case of daiokanzoto by the both methods. Since the concentration of sennoside A was not high, it was considered that it could not be reflected in the total evaluation of the decoction color.

## Conclusions

Based on the results from this study, the IPCD method is comparable to the conventional method in terms of the color, and using IPCD method, the same or greater amounts of quantitative indicator ingredients of crude drugs in the decoction of daiokanzoto compared to the conventional method. Therefore, there are limitations to assessing the equivalence of decoctions from decoction color. It is suggested that IPCD method may be useful in clinical settings although some caution is needed. Although more research on the optimal amount of crude drugs for the IPCD method, including quantitative analysis of various compounds in other crude drugs, is needed, it is prudent to use the IPCD method for Kampo formula decoction in clinical practice with a certain degree of caution.

## Electronic supplementary material

Below is the link to the electronic supplementary material.

Supplementary Material 1

## Data Availability

Not Applicable.

## List of abbreviations

HPLC: High performance liquid chromatography
IPCD: Immersing powdered crude drugs
JP18: Japanese Pharmacopoeia 18
