# Supplementary material for: Comparison of the conventional and immersing powdered crude drugs (IPCD) methods for color and extraction of quantitative indicator ingredients in the Kampo formula decoction of daiokanzoto
Source: J Pharm Health Care Sci. 2023 Jul 1;9:21. doi: 10.1186/s40780-023-00289-8 (PMC10314598; doi:10.1186/s40780-023-00289-8)
Supplement: Supplementary file 1 — Supplementary Material 1 [file 40780_2023_289_MOESM1_ESM.docx]

Dear Prof Masayoshi Koinuma

Editor, Journal of Pharmaceutical Health Care and Sciences

Thank you very much for reviewing our manuscript, “Comparison of the conventional and immersing powdered crude drugs (IPCD) methods for color and extraction of quantitative indicator ingredients in the Kampo formula decoction of daiokanzoto” and considering potential acceptance for publication. We corrected some minor problems that the reviewers had pointed out. (the changes were highlighted with red-letter text).

We hereby resubmit our manuscript, and thank you again for acceptance for publication in Journal of Pharmaceutical Health Care and Sciences.

Sincerely,

Kazumasa Naruhashi, PhD

Education and Research Center for Pharmacy Practice, Faculty of Pharmaceutical Sciences, Doshisha Women’s College of Liberal Arts

[knaruhas@dwc.doshisha.ac.jp](mailto:knaruhas@dwc.doshisha.ac.jp)

+81-774-65-8495

-------------Response to Reviewers-------------

Reviewer #1:

The authors well revised, but the reivised article still has minor problems.

1. In line 6, Background, the number of 148 Kampo formulations are approved as the ethical use. Total 294 formulations are approved by Japanese Government.

(Response) Thank you for pointing out the mistake. We corrected. (P3 L8)

2. In line 7, Background, "JP18" must be the abbreviation, and should be defined at the first appearance and add the reference.

(Response) We added “Japanese Pharmacopoeia 18th Edition” before the abbreviation of JP18. (P3 L8)

3. In the 1st line, the 4th paragraph of Background, and the 1st line, Materials, Japanese Pharmacopoeia registered the rhizome of other Rheum species as "rhubarb". And the format should be united with glycyrrhiza.

(Response) Explanations of "rhubarb" and “glycyrrhiza” were as described in JP18 English version.

(We have corrected in the manuscript of the previous version according to your previous suggestion.)

(P3 L26~29, P4 L3~4)

4. According to the package inseart of Tumura's daiokanzoto, the significant digit is 2 digits. In 5th line, materials, "4.00 g" and "2.00 g" are too accurate. If the authors measure rhubarb and glycyrrhiza for the experimental use, the authors can describe 3 digits numbers at the sampling section.

(Response) In 5th line, materials, "4.00 g" and "2.00 g" had been changed to 4.0 g and 2.0 g respectively in the manuscript of the previous version. (P4 L9)

According to your suggestion, we added the 3-digit numbers at the sampling sections. (P4 L15, L24~25)

5. In Fig. 3 and 5, if the authors conducted Student's t-test, the upper graph should be separated into 2 graphs. If one graph contains 4 groups, Student's t-test must not be accepted.

(Response) Each of the upper graphs of Figs 3 & 5 had been separated into 2 graphs in the manuscript of the previous version according to your previous suggestion. (Figure 3, Figure 5)

Reviewer #3:

All the points raised by the reviewers were addressed, and the context was revised appropriately. This reviewer points out just one point. The response to the 3rd comments of the reviewer 3 (the description of Kampo background, P3 L11-12), needs to the citation of  the PMDA site of 18JP in English Version (<https://www.pmda.go.jp/english/rs-sb-std/standards-development/jp/0029.html>). This is just a minor editorail suggestion.

(Response) We have sited the PMDA site in the manuscript of the previous version according to your suggestion. (P11 Ref #2)
